# Opportunities for opioid overdose prediction: building a population health approach

**DOI:** 10.1016/S2589-7500(22)00097-8

**Published:** 2022-06

**Authors:** Bennett Allen, Magdalena Cerdá

**Affiliations:** Center for Opioid Epidemiology and Policy, Department of Population Health, New York University Grossman School of Medicine, New York, 10016, USA

The opioid overdose epidemic remains a profound public health crisis in the USA. In recent years, the introduction of potent synthetic opioids to the illicit drug supply and the disturbances to the health care and substance use disorder treatment systems during the COVID-19 pandemic have accelerated overdose deaths.^1,2^ To meet these challenges, health-care professionals need new strategies. Predictive analytics could guide clinical decision making and inform health-care resource targeting.

In a prognostic modelling study published in *The Lancet Digital Health*, Wei-Hsuan Lo-Ciganic and colleagues present the development and validation of a machine-learning algorithm to predict opioid overdose among Medicaid beneficiaries.^3^ This study builds on a growing body of work at the intersection of medicine and data science—to which Lo-Ciganic and colleagues are leading contributors^4–6^—showing the utility of machine learning to inform clinical care for diverse patients with opioid use disorder or at risk for opioid overdose.

The authors use data from 958 278 patients in Pennsylvania and 391 959 patients in Arizona with histories of opioid analgesic prescription. They restrict their sample to patients with opioid prescription histories, as clinicians might more efficiently focus overdose prevention interventions to this group compared with a general patient population. The study aimed to build a model using data from one state and validate it using data from a different state to demonstrate the portability of short-run clinical overdose prediction.

The authors applied several commonly used classification models to their data. Results indicate that the authors’ best performing model predicted future patient opioid overdose risk with high discrimination ability. Findings from external validation of their gradient boosting machines model showed that the model performed well in different time periods within the same state (C-statistic of 0·841 [95% CI 0·835–0·847] for Pennsylvania 2013–16, n=639 693; and 0·828 [0·822–0·834] for Pennsylvania 2017–18, n=318 585) and in a different state (0·817 [0·807–0·826] for Arizona 2015–17).

This portable tool for providers who treat Medicaid populations fills a pressing gap in the literature.^3^ The authors notably predict patient opioid overdose risk with high predictive performance over a short-run period. Prediction across an abbreviated, 3-month risk period has the potential for strong clinical effect. It can inform provider strategies to engage patients in effective medication treatment for opioid use disorder, increase co-prescription of naloxone with opioids or benzodiazepines, and refer patients to ancillary social and harm reduction services.

Additionally, the authors’ validation using Medicaid data from two states with distinct demographic and drug policy profiles is promising. Given the breadth of Medicaid in the USA, a portable overdose risk prediction model using these data presents a novel clinical opportunity for state and federal policy makers. However, further research should assess performance using data capturing risk after the COVID-19 pandemic and in regional contexts with drug supply and patient profiles that differ from the Northeast (Pennsylvania) and Southwest (Arizona), such as South, Midwest, and Pacific Coast. Likewise, researchers must consider the social and ethical implications of such work, as opioid overdose increasingly has been criminalised across the USA in the last several years.^7^ It is crucial that clinical algorithms guide health care and social service interventions without secondary harm.

Latest advances in spatiotemporal machine learning have further shown the capacity for small-area prediction of opioid overdose mortality risk.^8^ As such, we see value in the use of predictive analytics for public health decision support. In the COVID-19 era more than ever, accurate and proactive resource targeting to high-risk geographies can help practitioners maximise preventive impact. Past work has illustrated the capacity for machine learning to identify characteristics of neighborhoods with elevated opioid overdose risk,^9^ and researchers are testing the use of predictive analytics for public health decision support.^10^ However, broader attention to prediction for population prevention is needed to realise the potential of machine learning in public health.

Clinical and population health researchers have much to learn from one another regarding the use of machine learning to improve health outcomes. We commend Lo-Ciganic and colleagues for their model, which holds potential to prevent opioid overdose at the individual level.^3^ Ending the opioid overdose epidemic is possible, and the shared toolkits of medicine, epidemiology, and data science have the potential to bend the curve through innovation and interdisciplinary engagement.
